# Supplementary material for: Reach, implementation fidelity, and safety of bubble continuous positive airway pressure (bCPAP) therapy in children with severe pneumonia in Pakistan
Source: PLOS Glob Public Health. 2026 Jun 15;6(6):e0006232. doi: 10.1371/journal.pgph.0006232 (PMC13268127; doi:10.1371/journal.pgph.0006232)
Supplement: S1 File — (PDF) [file pgph.0006232.s002.pdf]

Data Dictionary Codebook

Severe PNA epi study (Data collection 2) (PID: 2226)

07/07/2025 2:28am

Instruments

Instrument	Form Name	Events
Eligibility	eligibility	day_0_arm_1
Researcher And Participant Info	researcher_and_participant_info	day_0_arm_1
Demographics	demographics	day_0_arm_1
Initial clinical presentation	initial_clinical_presentation	day_0_arm_1
Initial physical exam	initial_physical_exam	day_0_arm_1
bCPAP management and implementation outcomes	bcpap_management_and_implementation_outcomes	day_0_arm_1
Adjunct therapies	adjunct_therapies	day_0_arm_1
Diagnostics	diagnostics	day_0_arm_1
Clinical outcomes	clinical_outcomes	day_0_arm_1
Researcher and Participant Info (subsequent day)	researcher_and_participant_info_subsequent_day	day_1_arm_1<br>day_2_arm_1<br>day_3_arm_1<br>day_4_arm_1<br>day_5_arm_1<br>day_6_arm_1<br>day_7_arm_1<br>day_8_arm_1<br>day_9_arm_1<br>day_10_arm_1<br>day_11_arm_1<br>day_12_arm_1<br>day_13_arm_1<br>day_14_arm_1<br>day_15_arm_1<br>day_16_arm_1<br>day_17_arm_1<br>day_18_arm_1<br>day_19_arm_1<br>day_20_arm_1<br>day_21_arm_1<br>day_22_arm_1<br>day_23_arm_1<br>day_24_arm_1<br>day_25_arm_1<br>day_26_arm_1<br>day_27_arm_1<br>day_28_arm_1<br>day_29_arm_1<br>day_30_arm_1
Current clinical condition (subsequent day)	current_clinical_condition_subsequent_day	day_1_arm_1<br>day_2_arm_1<br>day_3_arm_1<br>day_4_arm_1<br>day_5_arm_1<br>day_6_arm_1<br>day_7_arm_1<br>day_8_arm_1<br>day_9_arm_1<br>day_10_arm_1<br>day_11_arm_1<br>day_12_arm_1<br>day_13_arm_1<br>day_14_arm_1<br>day_15_arm_1<br>day_16_arm_1<br>day_17_arm_1<br>day_18_arm_1<br>day_19_arm_1<br>day_20_arm_1<br>day_21_arm_1<br>day_22_arm_1<br>day_23_arm_1<br>day_24_arm_1<br>day_25_arm_1<br>day_26_arm_1<br>day_27_arm_1

Instruments		
		day_28_arm_1<br>day_29_arm_1<br>day_30_arm_1
Physical exam (subsequent day)	physical_exam_subsequent_day	day_1_arm_1<br>day_2_arm_1<br>day_3_arm_1<br>day_4_arm_1<br>day_5_arm_1<br>day_6_arm_1<br>day_7_arm_1<br>day_8_arm_1<br>day_9_arm_1<br>day_10_arm_1<br>day_11_arm_1<br>day_12_arm_1<br>day_13_arm_1<br>day_14_arm_1<br>day_15_arm_1<br>day_16_arm_1<br>day_17_arm_1<br>day_18_arm_1<br>day_19_arm_1<br>day_20_arm_1<br>day_21_arm_1<br>day_22_arm_1<br>day_23_arm_1<br>day_24_arm_1<br>day_25_arm_1<br>day_26_arm_1<br>day_27_arm_1<br>day_28_arm_1<br>day_29_arm_1<br>day_30_arm_1
bCPAP management and implementation outcomes (subsequent day)	bcpap_management_and_implementation_outcomes_subse	day_1_arm_1<br>day_2_arm_1<br>day_3_arm_1<br>day_4_arm_1<br>day_5_arm_1<br>day_6_arm_1<br>day_7_arm_1<br>day_8_arm_1<br>day_9_arm_1<br>day_10_arm_1<br>day_11_arm_1<br>day_12_arm_1<br>day_13_arm_1<br>day_14_arm_1<br>day_15_arm_1<br>day_16_arm_1<br>day_17_arm_1<br>day_18_arm_1<br>day_19_arm_1<br>day_20_arm_1<br>day_21_arm_1<br>day_22_arm_1<br>day_23_arm_1<br>day_24_arm_1<br>day_25_arm_1<br>day_26_arm_1<br>day_27_arm_1<br>day_28_arm_1<br>day_29_arm_1<br>day_30_arm_1
Adjunct therapies (subsequent day)	adjunct_therapies_subsequent_day	day_1_arm_1<br>day_2_arm_1<br>day_3_arm_1<br>day_4_arm_1<br>day_5_arm_1<br>day_6_arm_1<br>day_7_arm_1<br>day_8_arm_1<br>day_9_arm_1<br>day_10_arm_1<br>day_11_arm_1<br>day_12_arm_1<br>day_13_arm_1<br>day_14_arm_1

Instruments		
		day_15_arm_1<br>day_16_arm_1<br>day_17_arm_1<br>day_18_arm_1<br>day_19_arm_1<br>day_20_arm_1<br>day_21_arm_1<br>day_22_arm_1<br>day_23_arm_1<br>day_24_arm_1<br>day_25_arm_1<br>day_26_arm_1<br>day_27_arm_1<br>day_28_arm_1<br>day_29_arm_1<br>day_30_arm_1
Diagnostics (subsequent day)	diagnostics_subsequent_day	day_1_arm_1<br>day_2_arm_1<br>day_3_arm_1<br>day_4_arm_1<br>day_5_arm_1<br>day_6_arm_1<br>day_7_arm_1<br>day_8_arm_1<br>day_9_arm_1<br>day_10_arm_1<br>day_11_arm_1<br>day_12_arm_1<br>day_13_arm_1<br>day_14_arm_1<br>day_15_arm_1<br>day_16_arm_1<br>day_17_arm_1<br>day_18_arm_1<br>day_19_arm_1<br>day_20_arm_1<br>day_21_arm_1<br>day_22_arm_1<br>day_23_arm_1<br>day_24_arm_1<br>day_25_arm_1<br>day_26_arm_1<br>day_27_arm_1<br>day_28_arm_1<br>day_29_arm_1<br>day_30_arm_1
Clinical outcomes (subsequent day)	clinical_outcomes_subsequent_day	day_1_arm_1<br>day_2_arm_1<br>day_3_arm_1<br>day_4_arm_1<br>day_5_arm_1<br>day_6_arm_1<br>day_7_arm_1<br>day_8_arm_1<br>day_9_arm_1<br>day_10_arm_1<br>day_11_arm_1<br>day_12_arm_1<br>day_13_arm_1<br>day_14_arm_1<br>day_15_arm_1<br>day_16_arm_1<br>day_17_arm_1<br>day_18_arm_1<br>day_19_arm_1<br>day_20_arm_1<br>day_21_arm_1<br>day_22_arm_1<br>day_23_arm_1<br>day_24_arm_1<br>day_25_arm_1<br>day_26_arm_1<br>day_27_arm_1<br>day_28_arm_1<br>day_29_arm_1<br>day_30_arm_1

Events	
**Event Name**	**Unique event name**
Day 0	day_0_arm_1
Day 1	day_1_arm_1
Day 2	day_2_arm_1
Day 3	day_3_arm_1
Day 4	day_4_arm_1
Day 5	day_5_arm_1
Day 6	day_6_arm_1
Day 7	day_7_arm_1
Day 8	day_8_arm_1
Day 9	day_9_arm_1
Day 10	day_10_arm_1
Day 11	day_11_arm_1
Day 12	day_12_arm_1
Day 13	day_13_arm_1
Day 14	day_14_arm_1
Day 15	day_15_arm_1
Day 16	day_16_arm_1
Day 17	day_17_arm_1
Day 18	day_18_arm_1
Day 19	day_19_arm_1
Day 20	day_20_arm_1
Day 21	day_21_arm_1
Day 22	day_22_arm_1
Day 23	day_23_arm_1
Day 24	day_24_arm_1
Day 25	day_25_arm_1
Day 26	day_26_arm_1
Day 27	day_27_arm_1
Day 28	day_28_arm_1
Day 29	day_29_arm_1
Day 30	day_30_arm_1

#	Variable / Field Name	Field Label<br>*Field Note*	Field Attributes (Field Type, Validation, Choices, Calculations, etc.)
Instrument: **Eligibility** (eligibility)			
1	[record_id]	Record ID	text
2	[ra_name]	Data collector name	dropdown, Required, Identifier<br>1 Ameera<br>2 Haania<br>3 Perah<br>4 Uzair
3	[age_elig]	Age 1-59 months?	yesno, Required<br>1 Yes<br>0 No
4	[time_now]	Current date and time	text (datetime_dmy), Required
5	[time_ed_present]	Date and time of initial presentation to ED	text (datetime_dmy, Min: [time_now]), Required
6	[resp_elig]	Which respiratory symptoms did the child present with?	radio, Required

			1 Cough<br>2 Difficulty breathing<br>3 Both cough and difficulty breathing<br>4 Neither
7	[hypox_elig]	SpO2< 90%? (from ED initial vital signs on room air)	yesno, Required<br>1 Yes<br>0 No
8	[cyanosis_elig]	Central cyanosis? (from medical chart review from initial ED presentation)	yesno, Required<br>1 Yes<br>0 No
9	[distress_elig]	Presented in severe respiratory distress? (e.g., grunting, very severe chest indrawing; based on chart review from initial ED presentation)	yesno, Required<br>1 Yes<br>0 No
10	[ds4_elig]	Vomiting everything? (ask caregiver about symptoms before ED presentation)	yesno, Required<br>1 Yes<br>0 No
11	[ds1_elig]	Inability to breastfeed or drink? (ask caregiver about symptoms before ED presentation)	yesno, Required<br>1 Yes<br>0 No
12	[ds2_elig]	Lethargy or unconscious? (from medical chart from initial ED presentation - do not just ask caregiver)	yesno, Required<br>1 Yes<br>0 No
13	[ds3_elig]	Convulsions? (ask caregiver about symptoms before ED presentation)	yesno, Required<br>1 Yes<br>0 No
14	[consent_elig]<br>Show the field ONLY if: [age_elig] = '1' and datediff ([time_now], [time_ed_present], 'm') < 1440 and ([resp_elig] = '1' or [resp_elig] = '2' or [resp_elig] = '3') and ([hypox_elig] = '1' or [cyanosis_elig] = '1' or [distress_elig] = '1' or [ds1_elig] = '1' or [ds2_elig] = '1' or [ds3_elig] = '1' or [ds4_elig] = '1')	This child has severe pneumonia and is eligible for the study. Written consent obtained?	yesno, Required<br>1 Yes<br>0 No
15	[not_eligible]<br>Show the field ONLY if: [age_elig] = '0' or datediff([time_now], [time_ed_present], 'm') >= 1440 or [resp_elig] = '4' or ([hypox_elig] = '0' and [cyanosis_elig] = '0' and [distress_elig] = '0' and [ds1_elig] = '0' and [ds2_elig] = '0' and [ds3_elig] = '0' and [ds4_elig] = '0')	This child is not eligible for the study. Please stop here.	descriptive
16	[eligibility_complete]	Section Header: *Form Status*<br>Complete?	dropdown<br>0 Incomplete<br>1 Unverified

			2 Complete
**Instrument: Researcher And Participant Info (researcher_and_participant_info)**			
17	[site]	Study site	dropdown, Required<br>1 Aga Khan University Hospital<br>2 Abbasi Shaheed
18	[participant_id]	Participant ID	text, Required
19	[mrn]	Child MRN	text, Required, Identifier
20	[mrn_confirm]	Confirm Child MRN	text, Required, Identifier
21	[child_name]	Child name	text, Required, Identifier
22	[parent_name]	Parent name	text, Required, Identifier
23	[child_dob]	Child date of birth	text (date_dmy, Max: today), Required, Identifier
24	[child_age]	Child age (months)	calc<br>Calculation: rounddown(datediff([child_dob], [time_now], 'M'))
25	[researcher_and_participant_info_complete]	Section Header: *Form Status*<br>Complete?	dropdown<br>0 Incomplete<br>1 Unverified<br>2 Complete
**Instrument: Demographics (demographics)**			
26	[demo_intro]	The information below should be obtained from the caregiver and supplemented with medical records (e.g., for child past medical history) as needed.	descriptive
27	[caregiver_age]	Caregiver age (years)	text (integer, Min: 10, Max: 110), Required
28	[caregiver_sex]	Caregiver sex	radio, Required<br>0 Female<br>1 Male<br>2 Other
29	[caregiver_marital]	Caregiver marital status	dropdown, Required<br>0 Married<br>1 Single<br>2 Divorced<br>3 Widowed
30	[caregiver_educ]	Caregiver educational level	dropdown, Required<br>0 No school<br>1 Some primary school<br>2 Completed primary school<br>3 Some secondary school<br>4 Completed secondary school<br>5 Some university<br>6 Completed university<br>7 Advanced degree
31	[language]	Caregiver preferred language	radio, Required<br>1 Urdu<br>2 Sindhi<br>3 Balochi<br>4 Pashto

			5 Punjabi<br>6 English<br>7 Other
32	[other_language]<br>Show the field ONLY if:<br>[language] = '7'	Specify other preferred language:	text, Required
33	[household_income]	Household monthly income (PKR)	radio, Required<br>0 < 50,000<br>1 50,000-100,000<br>2 >100,000
34	[home_location]	Home location	text, Required
35	[child_total]	Caregiver number of children	text (integer, Min: 0), Required
36	[child_no]<br>Show the field ONLY if:<br>[child_total] > 1	Child birth order (Must be an integer; 1 = oldest, 2 = second oldest, 3 = third oldest, etc.)	text (integer), Required
37	[child_sex]	Child sex	radio, Required<br>0 Female<br>1 Male<br>2 Other
38	[child_pmh]	Child past medical problems (from medical chart and asking caregiver)	text, Required
39	[child_weight]	Child weight (kg) (from medical chart)	text (number), Required
40	[demographics_complete]	Section Header: *Form Status*<br>Complete?	dropdown<br>0 Incomplete<br>1 Unverified<br>2 Complete
**Instrument: Initial clinical presentation (initial_clinical_presentation)**			
41	[duration_sx]	Duration of symptoms (in days) (Based on caregiver report. If symptoms started today, enter '0.')	text (integer, Min: 0), Required
42	[prior_care]	Did you seek medical care for your child's illness prior to coming to this hospital? (Based on caregiver report)	yesno, Required<br>1 Yes<br>0 No
43	[referral]	Did a healthcare professional tell you to bring your child to this hospital?	yesno, Required<br>1 Yes<br>0 No
44	[prior_location]	Where was child immediately before coming to this hospital? (Based on caregiver report)	radio, Required<br>0 Child's home<br>1 Relative's home<br>2 Outpatient clinic<br>3 Different hospital<br>4 Other
45	[prior_location_other]<br>Show the field ONLY if:<br>[prior_location] = '4'	Please specify other location prior to coming to hospital:	text, Required
46	[transport]	How did your child come to this hospital? (Based on caregiver report)	radio, Required

			1 Private vehicle (car, rickshaw, motorcycle, bicycle, etc.)<br>2 Public transport<br>3 On foot<br>4 Ambulance<br>5 Other
47	[transport_other]<br><br>Show the field ONLY if:<br>[transport] = '5'	Please specify other method of transportation to this hospital:	text, Required
48	[clinical_ed_presentation]	All these fields are based on the first documented physical exams/vital signs in the Emergency Department.	descriptive
49	[resp_distress]	How was the respiratory distress described (in chart) on ED presentation?	radio, Required<br>0 None<br>1 Mild<br>2 Moderate<br>3 Severe<br>4 Not mentioned/unknown
50	[initial_temp]	Initial temperature (C)	text (number, Min: 30, Max: 45), Required
51	[initial_hr]	Initial heart rate	text (integer, Min: 0, Max: 250), Required
52	[initial_rr]	Initial respiratory rate (breaths per min)	text (integer), Required
53	[initial_spo2]	Initial SpO2 (%) (while on room air)	text (integer), Required
54	[initial_sbp]	Initial systolic blood pressure	text (integer), Required
55	[initial_dbp]	Initial diastolic blood pressure	text (integer), Required
56	[initial_clinical_presentation_complete]	Section Header: *Form Status*<br>Complete?	dropdown<br>0 Incomplete<br>1 Unverified<br>2 Complete
**Instrument: Initial physical exam (initial_physical_exam)**			
57	[intro_initial_pe]	For this section, please record your current physical exam of the child.	descriptive
58	[muac]	Mid upper arm circumference (cm, to nearest 0.1cm)	text (number), Required
59	[rr_pe]	Respiratory rate (using stopwatch, count for 1 minute)	text (integer), Required
60	[spo2_pe]	SpO2 (%)	text, Required
61	[spo2_low]	Lowest SpO2 (%) recorded since presentation	text (integer, Min: 0, Max: 100), Required
62	[accessory_pe]	Use of accessory muscles?	radio, Required<br>0 Mild to no use of accessory muscles. Mild to no retractions or nasal flaring on inspiration<br>1 Moderate intercostal retractions, mild to moderate use of accessory muscles, nasal flaring.<br>2 Severe intercostal and substernal retractions, nasal flaring
63	[ms_pe]	Mental status?	radio, Required<br>0 Normal to Mildly irritable<br>1 Irritable, agitated, restless<br>2 Lethargic
64	[color_pe]	Color?	radio, Required

			0 Normal<br>1 Pale to normal<br>2 Cyanotic or dusky
65	[auscultation_pe]	Auscultation?	radio, Required<br>0 Good air movement, Expiratory scattered wheezing or loose rales/crackles<br>1 Depressed air movement, inspiratory and expiratory wheezes or rales/crackles<br>2 Diminished or absent breath sounds, severe wheezing or rales/crackles or marked prolonged expiration
66	[initial_physical_exam_complete]	Section Header: *Form Status*<br>Complete?	dropdown<br>0 Incomplete<br>1 Unverified<br>2 Complete
**Instrument: bCPAP management and implementation outcomes (bcpap_management_and_implementation_outcomes)**			
67	[bcpap]	Did child receive bCPAP? (based on chart review at any time since initial ED presentation)	yesno, Required<br>1 Yes<br>0 No
68	[bcpap_device]<br>Show the field ONLY if:<br>[bcpap] = '1'	bCPAP device type	radio, Required<br>0 Homemade bCPAP<br>1 Vayu bCPAP device<br>2 Other
69	[bcpap_device_other]<br>Show the field ONLY if:<br>[bcpap_device] = '2'	Please specify other bCPAP device type:	text, Required
70	[bcpap_start]<br>Show the field ONLY if:<br>[bcpap] = '1'	Was bCPAP started within 1 hour of initial presentation? "Yes" only if bCPAP start time < 1 hour affter initial presentation documented in chart If not documented, select "Don't know"	radio, Required<br>0 No<br>1 Yes<br>99 Don't know
71	[bcpap_start_time]<br>Show the field ONLY if:<br>[bcpap] = '1'	bCPAP start time (if known from medical chart)	text (datetime_dmy, Min: [time_ed_present]), Required
72	[bcpap_current]<br>Show the field ONLY if:<br>[bcpap] = '1'	Does the child remain on bCPAP now?	radio, Required<br>0 No<br>1 Yes
73	[bcpap_stop_time]<br>Show the field ONLY if:<br>[bcpap] = '1' and [bcpap_current] = '0'	bCPAP stop time (if known from medical chart)	text (datetime_dmy, Min: [bcpap_start_time]), Required
74	[bcpap_pressure]<br>Show the field ONLY if:<br>[bcpap_current] = '1'	Current bCPAP pressure	text (integer), Required
75	[bcpap_fio2]<br>Show the field ONLY if:<br>[bcpap_current] = '1'	Current bCPAP FiO2 (enter 100% oxygen is using medical oxygen without blender and 21% if using medical air)	text (integer), Required
76	[bubbles]<br>Show the field ONLY if:	Is the bCPAP currently bubbling?	yesno, Required<br>1 Yes

	[bcpap_current] = '1'		0 No
77	[diet]	Current diet order (from medical chart review)	radio, Required<br>0 NPO<br>1 Nasogastric or orogastric tube feeds<br>2 Oral feeds (PO)<br>3 Other
78	[other_diet]<br>Show the field ONLY if:<br>[diet] = '3'	Please describe the other diet order	text, Required
79	[po_feeds]<br>Show the field ONLY if:<br>[bcpap] = '1'	Did the child receive feeds by mouth (ordered by doctor) while on bCPAP since presentation? Check medical chart for order, confirm with RN and caregiver regarding feeding	radio, Required<br>0 No<br>1 Yes<br>99 Don't know
80	[ngt]	Was a gastric (nasal or oral) tube placed since presentation? Examine patient and ask RN	radio, Required<br>0 No<br>1 Yes<br>99 Don't know
81	[ng_feeds]<br>Show the field ONLY if:<br>[bcpap] = '1' and [ngt] = '1'	Did the child receive gastric feeds (ordered by doctor) while on bCPAP since presentation? Review medical chart and ask RN	radio, Required<br>0 No<br>1 Yes<br>99 Don't know
82	[caregiver_feeds]<br>Show the field ONLY if:<br>[bcpap] = '1'	Did the caregiver give child any additional feeds while on bCPAP since presentation? Normalize the statement and ask the caregiver ("Some families give their child additional food beyond what the doctor ordered. Is this something you have done while your child has been on bCPAP?")	radio, Required<br>0 No<br>1 Yes<br>99 Don't know
83	[disruption]<br>Show the field ONLY if:<br>[bcpap] = '1'	Have there been any unplanned disruptions to bCPAP therapy? (e.g., cannula falling out) Ask caregiver and RN separately. Give examples of disruptions (cannula falling out, child pulling cannula off, tubing disconnecting, bottle no longer bubbling)	radio, Required<br>0 No<br>1 Yes<br>99 Don't know
84	[disruption_reason]<br>Show the field ONLY if:<br>[disruption] = '1'	If yes, what caused the disruption? (based on RN/caregiver response)	text, Required
85	[disruption_id]<br>Show the field ONLY if:<br>[disruption] = '1'	Who identified that there was a disruption? (can select multiple answers; based on RN/caregiver report)	checkbox, Required<br>1 disruption_id___1 RN<br>2 disruption_id___2 Doctor<br>3 disruption_id___3 Caregiver<br>4 disruption_id___4 Other
86	[disruption_resolve]<br>Show the field ONLY if:<br>[disruption] = '1'	Who resolved the disruption? (can select multiple answers; based on RN/caregiver report)	checkbox, Required<br>1 disruption_resolve___1 RN<br>2 disruption_resolve___2 Doctor<br>3 disruption_resolve___3 Caregiver<br>4 disruption_resolve___4 Other
87	[disruption_duration]<br>Show the field ONLY if:<br>[disruption] = '1'	How long did the disruption last? (based on RN/caregiver response)	text, Required

88	[vitals]	How many times have vital signs been recorded since presentation? (review medical chart, include initial set of vital signs)	text (integer), Required
89	[hours_since_admit]	Current time (click "now")	text (datetime_dmy), Required
90	[monitor]	Is patient currently on a continuous monitor? (a multiparameter monitor measures multiple vital signs; a pulse oximeter only measures SpO2)	radio, Required<br>0 No<br>1 Yes (pulse ox only)<br>2 Yes (multiparameter monitor)
91	[bcpap_management_and_implementation_outcomes_complete]	Section Header: *Form Status*<br>Complete?	dropdown<br>0 Incomplete<br>1 Unverified<br>2 Complete
**Instrument: Adjunct therapies (adjunct_therapies)**			
92	[adjunct_intro]	Please indicate whether each of the following adjunctive therapies were given to the patient (since initial presentation to the ED). This is based on review of the medical chart.	descriptive
93	[cpt]	Chest physiotherapy (based on caregiver and/or RN report)	radio, Required<br>0 No<br>1 Yes<br>99 Don't know
94	[nebs]	Nebulized bronchodilator	radio, Required<br>0 No<br>1 Yes<br>99 Don't know
95	[abx]	Antibiotics	radio, Required<br>0 No<br>1 Yes<br>99 Don't know
96	[ivf]	IV fluids	radio, Required<br>0 No<br>1 Yes<br>99 Don't know
97	[antipyretics]	Antipyretics	radio, Required<br>0 No<br>1 Yes<br>99 Don't know
98	[vasoactives]	Vasoactive medications	radio, Required<br>0 No<br>1 Yes<br>99 Don't know
99	[steroids]	Corticosteroids	radio, Required<br>0 No<br>1 Yes<br>99 Don't know
100	[prbcs]	pRBC transfusion	radio, Required

			0 No<br>1 Yes<br>99 Don't know
101	[chest_tube]	Chest drain placement	radio, Required<br>0 No<br>1 Yes<br>99 Don't know
102	[surgery]	Need for pneumonia-related surgery (e.g., VATS)	radio, Required<br>0 No<br>1 Yes<br>99 Don't know
103	[adjunct_therapies_complete]	Section Header: *Form Status*<br>Complete?	dropdown<br>0 Incomplete<br>1 Unverified<br>2 Complete
**Instrument: Diagnostics (diagnostics)**			
104	[intro_diagnostics]	This form asks for whether the patient had the following diagnostics done since initial presentation to the ED based on review of the medical chart.	descriptive
105	[cxr]	Was a chest radiograph done?	radio, Required<br>0 No<br>1 Yes<br>99 Don't know
106	[cxr_findings]<br>Show the field ONLY if:<br>[cxr] = '1'	Chest radiograph interpretation	text, Required
107	[culture]	Was a microbial culture obtained?	radio, Required<br>0 No<br>1 Yes<br>99 Don't know
108	[culture_date]<br>Show the field ONLY if:<br>[culture] = '1'	Culture date	text (date_dmy), Required
109	[culture_site]<br>Show the field ONLY if:<br>[culture] = '1'	Culture site	radio, Required<br>0 Sputum<br>1 Lower respiratory<br>2 Blood<br>3 Pleural fluid<br>4 Other<br>99 Don't know
110	[culture_result]<br>Show the field ONLY if:<br>[culture] = '1'	Culture result	text, Required
111	[culture_growth_date]<br>Show the field ONLY if:<br>[culture] = '1'	Culture date of growth	text (date_dmy), Required

112	[culture_2]	Was a 2nd microbial culture obtained?	radio, Required<br>0 No<br>1 Yes<br>99 Don't know
113	[culture_date_2]<br><br>Show the field ONLY if:<br>[culture_2] = '1'	Culture #2 date	text (date_dmy), Required
114	[culture_site_2]<br><br>Show the field ONLY if:<br>[culture_2] = '1'	Culture #2 site	radio, Required<br>0 Sputum<br>1 Lower respiratory<br>2 Blood<br>3 Pleural fluid<br>4 Other<br>99 Don't know
115	[culture_result_2]<br><br>Show the field ONLY if:<br>[culture_2] = '1'	Culture #2 result	text, Required
116	[culture_growth_date_2]<br><br>Show the field ONLY if:<br>[culture_2] = '1'	Culture #2 date of growth	text (date_dmy), Required
117	[other_cultures]	Other microbial culture results?	text
118	[diagnostics_complete]	Section Header: *Form Status*<br>Complete?	dropdown<br>0 Incomplete<br>1 Unverified<br>2 Complete
**Instrument: Clinical outcomes (clinical_outcomes)**			
119	[initial_dx]	Initial diagnosis (based on chart review)	text, Required
120	[dispo]	Current child disposition	radio, Required<br>0 Stepdown unit/special care area<br>1 General ward<br>2 PICU<br>3 Emergency department<br>4 Discharged to home<br>5 Transferred to another hospital<br>6 Left against medical advice<br>7 Died
121	[resp_support]<br><br>Show the field ONLY if:<br>[dispo] = '0' or [dispo] = '1' or [dispo] = '2' or [dispo] = '3'	Current respiratory support	radio, Required<br>0 bCPAP<br>1 Room air<br>2 Low-flow oxygen<br>3 High-flow oxygen<br>4 Mechanical non-invasive positive pressure ventilation (CPAP, BiPAP)<br>5 Invasive mechanical ventilation
122	[aspiration]	Has an aspiration event occurred since ED presentation? -Review medical chart for any documented aspiration events -Ask caregiver if they have witnessed child	radio, Required<br>0 No

		vomit/bring stomach contents into their mouth followed by immediate coughing/gagging -Ask RN and medical team if patient has had a suspected aspiration event	1 Yes<br>99 Don't know
123	[ptx]	Has a pneumothorax occurred since ED presentation? (based on medical chart review)	radio, Required<br>0 No<br>1 Yes<br>99 Don't know
124	[notes]	Please add any additional notes here	notes
125	[clinical_outcomes_complete]	Section Header: *Form Status*<br>Complete?	dropdown<br>0 Incomplete<br>1 Unverified<br>2 Complete
**Instrument: Researcher and Participant Info (subsequent day) (researcher_and_participant_info_subsequent_day)**			
126	[collector_name_subseq]	Data collector name	dropdown, Required, Identifier<br>1 Ameera<br>2 Haania<br>3 Perah<br>4 Uzair
127	[collection_time_subseq]	Date and time of data collection	text (datetime_dmy), Required
128	[location_subseq]	Current child location	radio, Required<br>0 Stepdown unit/special care area<br>1 General ward<br>2 PICU<br>3 Emergency department<br>4 Other
129	[ward_subseq]	Ward name (e.g., B0, D0, P0, PZP, PICU, etc.)	text
130	[other_location_subseq]<br>Show the field ONLY if:<br>[location_subseq] = '4'	If other, please specify location	text, Required
131	[researcher_and_participant_info_subsequent_day_complete]	Section Header: *Form Status*<br>Complete?	dropdown<br>0 Incomplete<br>1 Unverified<br>2 Complete
**Instrument: Current clinical condition (subsequent day) (current_clinical_condition_subsequent_day)**			
132	[dispo_subseq]	Current child disposition	radio, Required<br>0 Stepdown unit/special care area<br>1 General ward<br>2 ICU<br>3 Emergency department<br>4 Discharged to home<br>5 Transferred to another hospital<br>6 Left against medical advice<br>7 Died
133	[dod]<br>Show the field ONLY if:	Date of discharge/transfer/death	text (datetime_dmy), Required

	[dispo_subseq] = '4' or [dispo_subseq] = '5' or [dispo_subseq] = '6' or [dispo_subseq] = '7'		
134	[reason_tx]<br><br>Show the field ONLY if:<br>[dispo_subseq] = '5' or [dispo_subseq] = '6'	Reason for transfer or leaving AMA	text, Required
135	[cod]<br><br>Show the field ONLY if:<br>[dispo_subseq] = '7'	Cause of death:	text, Required
136	[discharge_dx]<br><br>Show the field ONLY if:<br>[dispo_subseq] = '4' or [dispo_subseq] = '5' or [dispo_subseq] = '6' or [dispo_subseq] = '7'	Discharge/final diagnosis (please also add secondary diagnoses)	text, Required
137	[resp_support_subseq]<br><br>Show the field ONLY if:<br>[dispo_subseq] = '0' or [dispo_subseq] = '1' or [dispo_subseq] = '2' or [dispo_subseq] = '3'	Current respiratory support	radio, Required<br>0 bCPAP<br>1 Room air<br>2 Low-flow oxygen<br>3 High-flow oxygen<br>4 Mechanical non-invasive positive pressure ventilation<br>5 Invasive mechanical ventilation
138	[resp_support_tx]<br><br>Show the field ONLY if:<br>[dispo_subseq] = '6' or [dispo_subseq] = '5' or [dispo_subseq] = '4'	Most recent respiratory support prior to discharge/transfer	radio, Required<br>0 bCPAP<br>1 Room air<br>2 Low-flow oxygen<br>3 High-flow oxygen<br>4 Mechanical non-invasive positive pressure ventilation<br>5 Invasive mechanical ventilation
139	[bcpap_start_subseq]<br><br>Show the field ONLY if:<br>([previous-event-name]='day_0_arm_1' and ([day_0_arm_1][bcpap_current]='0' or [day_0_arm_1][bcpap]='0') and [day_1_arm_1][resp_support_subseq]='0') or ([resp_support_subseq][previous-instance]<>'0' and [resp_support_subseq][current-instance]='0')	bCPAP start time	text (datetime_dmy), Required
140	[bcpap_stop_subseq]<br><br>Show the field ONLY if:<br>([previous-event-name] = 'day_0_arm_1' and [day_0_arm_1][bcpap_current]='1' and [day_1_arm_1][resp_support_subseq]<>'0') or ([resp_support_subseq][previous-instance]='0' and [resp_sup	bCPAP stop time	text (datetime_dmy), Required

	port_subseq][current-instance]<>'0')		
141	[current_clinical_condition_subsequent_day_complete]	Section Header: *Form Status*<br>Complete?	dropdown<br>0 Incomplete<br>1 Unverified<br>2 Complete
**Instrument: Physical exam (subsequent day) (physical_exam_subsequent_day)**			
142	[intro_initial_pe_subseq]	For this section, please record your current physical exam of the child.	descriptive
143	[hr_subseq]	Heart rate (bpm)	text (integer), Required
144	[rr_pe_subseq]	Respiratory rate (using stopwatch)	text (integer), Required
145	[spo2_pe_subseq]	SpO2 (%)	text, Required
146	[spo2_low_subseq]	Lowest recorded SpO2 (%) since last survey	text (integer, Min: 0, Max: 100), Required
147	[accessory_pe_subseq]	Use of accessory muscles?	radio, Required<br>0 Mild to no use of accessory muscles. Mild to no retractions or nasal flaring on inspiration<br>1 Moderate intercostal retractions, mild to moderate use of accessory muscles, nasal flaring.<br>2 Severe intercostal and substernal retractions, nasal flaring
148	[ms_pe_subseq]	Mental status?	radio, Required<br>0 Normal to Mildly irritable<br>1 Irritable, agitated, restless<br>2 Lethargic
149	[color_pe_subseq]	Color?	radio, Required<br>0 Normal<br>1 Pale to normal<br>2 Cyanotic or dusky
150	[auscultation_pe_subseq]	Auscultation?	radio, Required<br>0 Good air movement, Expiratory scattered wheezing or loose rales/crackles<br>1 Depressed air movement, inspiratory and expiratory wheezes or rales/crackles<br>2 Diminished or absent breath sounds, severe wheezing or rales/crackles or marked prolonged expiration
151	[physical_exam_subsequent_day_complete]	Section Header: *Form Status*<br>Complete?	dropdown<br>0 Incomplete<br>1 Unverified<br>2 Complete
**Instrument: bCPAP management and implementation outcomes (subsequent day) (bcpap_management_and_implementation_outcomes_subse)**			
152	[bcpap_device_subseq]<br>Show the field ONLY if:<br>[resp_support_subseq] = '0'	bCPAP device type	radio, Required<br>0 Homemade bCPAP<br>1 Vayu bCPAP device<br>2 Other
153	[bcpap_device_other_subseq]	Please specify other bCPAP device type	text, Required

	Show the field ONLY if: [bcpap_device_subseq] = '2'		
154	[pressure_subseq] Show the field ONLY if: [resp_support_subseq] = '0'	Current bCPAP pressure	text (integer), Required
155	[fio2_subseq] Show the field ONLY if: [resp_support_subseq] = '0'	Current bCPAP FiO2 (enter 100% oxygen if using medical oxygen without blender and 21% if using medical air)	text (integer), Required
156	[bubbles_subseq] Show the field ONLY if: [resp_support_subseq] = '0'	Is the bCPAP currently bubbling?	yesno, Required<br>1 Yes<br>0 No
157	[diet_subseq]	Current diet order (from medical chart review)	radio, Required<br>0 NPO<br>1 Nasogastric or orogastric feeds<br>2 Oral feeds (PO)<br>3 Other
158	[other_diet_subseq] Show the field ONLY if: [diet_subseq] = '3'	Please describe "other" diet order	text, Required
159	[po_feeds_subseq] Show the field ONLY if: ([previous-event-name]='day_0_arm_1' and [day_0_arm_1][bcpap_current]='1') or [resp_support_subseq][previous-instance]='0' or [resp_support_subseq]='0'	Did the child receive feeds by mouth (ordered by doctor) while on bCPAP since last survey? Check medical chart for order, confirm with RN and caregiver regarding feeding. Last survey refers to "current time" field in yesterday's bCPAP management and implementation outcomes form.	radio, Required<br>0 No<br>1 Yes<br>99 Don't know
160	[ngt_subseq]	Was a gastric (nasal or oral) tube placed since last survey? Examine patient and ask RN. Last survey refers to "current time" field in yesterday's bCPAP management and implementation outcomes form.	radio, Required<br>0 No<br>1 Yes<br>99 Don't know
161	[ng_feeds_subseq] Show the field ONLY if: ([previous-event-name]='day_0_arm_1' and [day_0_arm_1][bcpap_current]='1') or [resp_support_subseq][previous-instance]='0' or [resp_support_subseq]='0'	Did the child receive gastric feeds (ordered by doctor) while on bCPAP since last survey? Review medical chart and ask RN. Last survey refers to "current time" field in yesterday's bCPAP management and implementation outcomes form.	radio, Required<br>0 No<br>1 Yes<br>99 Don't know
162	[caregiver_feeds_subseq] Show the field ONLY if: ([previous-event-name]='day_0_arm_1' and [day_0_arm_1][bcpap_current]='1') or [resp_support_subseq][previous-instance]='0' or [resp_support_subseq]='0'	Did the caregiver give child any additional feeds while on bCPAP since last survey? Normalize the statement and ask the caregiver ("Some families give their child additional food beyond what the doctor ordered. Is this something you have done while your child has been on bCPAP?"). Last survey refers to "current time" field in yesterday's bCPAP management and implementation outcomes form.	radio, Required<br>0 No<br>1 Yes<br>99 Don't know
163	[disruption_subseq] Show the field ONLY if: ([previous-event-name]='day_0_arm_1' and [day_0_arm_1][bcpap_current]='1') or [resp_support_subseq][previ	Have there been any unplanned disruptions to bCPAP therapy since last survey? (e.g., cannula falling out) Ask caregiver and RN separately. Give examples of disruptions (cannula falling out, child pulling cannula off, tubing disconnecting, bottle no longer bubbling). Last survey	radio, Required<br>0 No<br>1 Yes<br>99 Don't know

	ous-instance]='0' or [resp_support_subseq]='0'	refers to "current time" field in yesterday's bCPAP management and implementation outcomes form.	
164	[disruption_reason_subseq]<br><br>Show the field ONLY if:<br>[disruption_subseq] = '1'	If yes, what caused the disruption? (based on RN/caregiver response)	text, Required
165	[disruption_id_subseq]<br><br>Show the field ONLY if:<br>[disruption_subseq] = '1'	Who identified that there was a disruption? (can select multiple answers; based on RN/caregiver report)	checkbox, Required<br>1 disruption_id_subseq___1 RN<br>2 disruption_id_subseq___2 Doctor<br>3 disruption_id_subseq___3 Caregiver<br>4 disruption_id_subseq___4 Other
166	[disruption_resolve_subseq]<br><br>Show the field ONLY if:<br>[disruption_subseq] = '1'	Who resolved the disruption? (can select multiple answers; based on RN/caregiver report)	checkbox, Required<br>1 disruption_resolve_subseq___1 RN<br>2 disruption_resolve_subseq___2 Doctor<br>3 disruption_resolve_subseq___3 Caregiver<br>4 disruption_resolve_subseq___4 Other
167	[disruption_duration_subseq]<br><br>Show the field ONLY if:<br>[disruption_subseq] = '1'	How long did the disruption last? (based on RN/caregiver response)	text, Required
168	[vitals_subseq]	How many times have vital signs been recorded since last survey? Please "current time" entered on bCPAP management and implementation outcomes page from yesterday's survey and count all times vital signs have been recorded since that time.	text (integer), Required
169	[current_time_subseq]	Current time (click "now")	text (datetime_dmy), Required
170	[monitor_subseq]	Is patient currently on a continuous monitor?	radio, Required<br>0 No<br>1 Yes (pulse ox only)<br>2 Yes (multiparameter monitor)
171	[bcpap_management_and_implementation_outcomes_subse_complete]	Section Header: *Form Status*<br>Complete?	dropdown<br>0 Incomplete<br>1 Unverified<br>2 Complete
**Instrument: Adjunct therapies (subsequent day) (adjunct_therapies_subsequent_day)**			
172	[adjunct_intro_subseq]	Please indicate whether each of the following adjunctive therapies were given to the patient (since last survey).	descriptive
173	[cpt_subseq]	Chest physiotherapy (based on caregiver and/or RN report)	radio, Required<br>0 No<br>1 Yes<br>99 Don't know
174	[nebs_subseq]	Nebulized bronchodilator	radio, Required<br>0 No<br>1 Yes<br>99 Don't know
175	[abx_subseq]	Antibiotics	radio, Required<br>0 No<br>1 Yes

			99 Don't know
176	[ivf_subseq]	IV fluids	radio, Required<br>0 No<br>1 Yes<br>99 Don't know
177	[antipyretics_subseq]	Antipyretics	radio, Required<br>0 No<br>1 Yes<br>99 Don't know
178	[vasoactives_subseq]	Vasoactive medications	radio, Required<br>0 No<br>1 Yes<br>99 Don't know
179	[steroids_subseq]	Corticosteroids	radio, Required<br>0 No<br>1 Yes<br>99 Don't know
180	[prbcs_subseq]	pRBC transfusion	radio, Required<br>0 No<br>1 Yes<br>99 Don't know
181	[chest_tube_subseq]	Chest drain placement	radio, Required<br>0 No<br>1 Yes<br>99 Don't know
182	[surgery_subseq]	Need for pneumonia-related surgery (e.g., VATS)	radio, Required<br>0 No<br>1 Yes<br>99 Don't know
183	[adjunct_therapies_subsequent_day_complete]	Section Header: *Form Status*<br>Complete?	dropdown<br>0 Incomplete<br>1 Unverified<br>2 Complete
**Instrument: Diagnostics (subsequent day) (diagnostics_subsequent_day)**			
184	[diagnostics_intro_subseq]	Please complete the following form for diagnostics obtained since the last survey.	descriptive
185	[cxr_subseq]	Was a chest radiograph done?	radio, Required<br>0 No<br>1 Yes<br>99 Don't know
186	[cxr_findings_subseq]<br>Show the field ONLY if:<br>[cxr_subseq] = '1'	Chest radiograph interpretation	text, Required
187	[culture_subseq]	Was a microbial culture obtained?	radio, Required<br>0 No

			1 Yes<br>99 Don't know
188	[culture_date_subseq]<br>Show the field ONLY if:<br>[culture_subseq] = '1'	Culture date	text (date_dmy), Required
189	[culture_site_subseq]<br>Show the field ONLY if:<br>[culture_subseq] = '1'	Culture site	radio, Required<br>0 Sputum<br>1 Lower respiratory<br>2 Blood<br>3 Pleural fluid<br>4 Other<br>99 Don't know
190	[culture_result_subseq]<br>Show the field ONLY if:<br>[culture_subseq] = '1'	Culture result	text, Required
191	[culture_growth_date_subseq]<br>Show the field ONLY if:<br>[culture_subseq] = '1'	Culture date of growth	text (date_dmy), Required
192	[past_culture_results]	New results for past cultures	text
193	[other_micro_results_subseq]	Other microbiologic results	text
194	[diagnostics_subsequent_day_complete]	Section Header: *Form Status*<br>Complete?	dropdown<br>0 Incomplete<br>1 Unverified<br>2 Complete
**Instrument: Clinical outcomes (subsequent day) (clinical_outcomes_subsequent_day)**			
195	[aspiration_subseq]	Aspiration event since last survey? -Review medical chart for any documented aspiration events -Ask caregiver if they have witnessed child vomit/bring stomach contents into their mouth followed by immediate coughing/gagging -Ask RN and medical team if patient has had a suspected aspiration event	radio, Required<br>0 No<br>1 Yes<br>99 Don't know
196	[ptx_subseq]	Pneumothorax since last survey? (based on medical chart review)	radio, Required<br>0 No<br>1 Yes<br>99 Don't know
197	[notes_subseq]	Please add any additional notes here	notes
198	[clinical_outcomes_subsequent_day_complete]	Section Header: *Form Status*<br>Complete?	dropdown<br>0 Incomplete<br>1 Unverified<br>2 Complete
