# Supplementary material for: Reach, implementation fidelity, and safety of bubble continuous positive airway pressure (bCPAP) therapy in children with severe pneumonia in Pakistan
Source: PLOS Glob Public Health. 2026 Jun 15;6(6):e0006232. doi: 10.1371/journal.pgph.0006232 (PMC13268127; doi:10.1371/journal.pgph.0006232)
Supplement: S1 Table — (DOCX) [file pgph.0006232.s004.docx]

**S1 Table. Child and caregiver characteristics for children who did and did not receive bCPAP, by study site.**

| **Study site** | **AKUH^a^ (N=110)** | | **ASH^b^ (N=55)** | | **Overall (N=165)** | | **Total** |
| --- | --- | --- | --- | --- | --- | --- | --- |
|  | No bCPAP | bCPAP | No bCPAP | bCPAP | No bCPAP | bCPAP |  |
|  | n = 65 | n = 45 | n = 12 | n = 43 | n = 77 | n = 88 | n = 165 |
| **Child characteristics, n (%)** | | | | | | | |
| **Age (mo), median (IQR)** | 13 (6-32) | 11 (6-16) | 9.5 (7-43) | 8 (6-17) | 13 (7-33) | 9 (6-16) | 10 (6-23) |
| **Sex** |  |  |  |  |  |  |  |
| Male | 37 (57) | 27 (60) | 5 (42) | 22 (51) | 42 (55) | 49 (56) | 91 (55) |
| Female | 28 (43) | 18 (40) | 7 (58) | 21 (49) | 35 (46) | 39 (44) | 74 (45) |
| **Chronic medical condition^a^** | 22 (34) | 12 (27) | 1 (8.3) | 7 (16) | 23 (30) | 19 (22) | 42 (26) |
| Recurrent pneumonia | 8 (12) | 7 (16) | 3 (25) | 4 (9.3) | 11 (14) | 11 (13) | 22 (13) |
| Asthma | 7 (11) | 1 (2.2) | 1 (8.3) | 0 (0.0) | 8 (10) | 1 (1.1) | 9 (5.5) |
| Congenital heart disease | 4 (6.2) | 4 (8.9) | 0 (0.0) | 2 (4.7) | 4 (5.2) | 6 (6.8) | 10 (6.1) |
| Prematurity | 2 (3.1) | 1 (2.2) | 0 (0.0) | 0 (0.0) | 2 (2.6) | 1 (1.1) | 3 (1.8) |
| Neurologic/developmental | 5 (7.7) | 3 (6.7) | 0 (0.0) | 3 (7.0) | 5 (6.5) | 6 (6.8) | 11 (6.7) |
| **Nutritional status** |  |  |  |  |  |  |  |
| Moderate malnutrition^d^ | 9 (14) | 8 (18) | 2 (17) | 8 (19) | 11 (14) | 16 (18) | 27 (16) |
| Severe malnutrition^d^ | 9 (14) | 11 (24) | 4 (33) | 17 (40) | 13 (17) | 28 (32) | 41 (25) |
| **Caregiver characteristics, n (%)** | | | | | | | |
| **Age (yrs), median (IQR)** | 31 (27-35) | 30 (28-34.5) | 27 (26-32.5) | 26 (22-30) | 31 (26-35) | 29 (25-32) | 30 (26-33.5) |
| **Sex** |  |  |  |  |  |  |  |
| Male | 20 (31) | 10 (22) | 1 (8.3) | 6 (14) | 21 (27) | 16 (18) | 37 (22) |
| Female | 45 (69) | 35 (78) | 11 (92) | 37 (86) | 56 (73) | 72 (82) | 128 (78) |
| **Highest educational level** |  |  |  |  |  |  |  |
| No school | 3 (4.6) | 3 (6.7) | 3 (25) | 10 (23) | 6 (7.8) | 13 (15) | 19 (12) |
| Some primary school | 6 (9.2) | 2 (4.4) | 1 (8.3) | 7 (16) | 7 (9.1) | 9 (10) | 16 (9.7) |
| Completed primary school | 7 (11) | 3 (6.7) | 4 (33) | 10 (23) | 11 (14) | 13 (15) | 24 (15) |
| Completed secondary school | 21 (32) | 11 (24) | 2 (17) | 16 (37) | 23 (30) | 27 (31) | 50 (30) |
| Completed university | 20 (31) | 17 (38) | 2 (17) | 0 (0.0) | 22 (29) | 17 (19) | 39 (24) |
| Advanced degree | 8 (12) | 9 (20.0) | 0 (0.0) | 0 (0.0) | 8 (10) | 9 (10) | 17 (10) |
| **Household monthly income** |  |  |  |  |  |  |  |
| <50,000 PKR | 11 (17) | 13 (29) | 10 (83) | 39 (91) | 21 (27) | 52 (59) | 73 (44) |
| 50,000-100,000 PKR | 20 (31) | 4 (8.9) | 2 (17) | 4 (9.3) | 22 (29) | 8 (9.1) | 30 (18) |
| >100,000 PKR | 30 (46) | 24 (53) | 0 (0.0) | 0 (0.0) | 30 (39) | 24 (27) | 54 (33) |
| Declined to answer | 4 (6.2) | 4 (8.9) | 0 (0.0) | 0 (0.0) | 4 (5.2) | 4 (4.6) | 8 (4.9) |

^a^AKUH: Aga Khan University Hospital

^b^ASH: Abbasi Shaheed Hospital

^c^Does not include recurrent pneumonia or recurrent acute infections without underlying chronic diagnosis.

^d^Moderate malnutrition refers to moderate acute malnutrition (11.5cm ≤ mid-upper arm circumference < 12.5cm) or moderate underweight (-3 ≤ weight-for-age z-score < -2). Severe malnutrition refers to severe acute malnutrition (mid-upper arm circumference < 11.5) or severe underweight (weight-for-age z-score < -3).
