# Supplementary material for: Reach, implementation fidelity, and safety of bubble continuous positive airway pressure (bCPAP) therapy in children with severe pneumonia in Pakistan
Source: PLOS Glob Public Health. 2026 Jun 15;6(6):e0006232. doi: 10.1371/journal.pgph.0006232 (PMC13268127; doi:10.1371/journal.pgph.0006232)
Supplement: S2 Table — (DOCX) [file pgph.0006232.s005.docx]

**S2 Table. Final disposition and mortality for children who did and did not receive bCPAP, by study site.**

| **Study site** | **AKU^a^ (n=110)** | | **ASH^b^ (n=55)** | | **Overall (n=165)** | | **Total** |
| --- | --- | --- | --- | --- | --- | --- | --- |
|  | **No bCPAP** | **bCPAP** | **No bCPAP** | **bCPAP** | **No bCPAP** | **bCPAP** |  |
|  | n = 65 | n = 45 | n = 12 | n = 43 | n = 77 | n = 88 | n = 165 |
| **Final disposition, n (%)** |  |  |  |  |  |  |  |
| Discharged to home | 55 (85) | 35 (78) | 4 (33) | 14 (33) | 59 (77) | 49 (56) | 108 (66) |
| Transferred to another hospital | 2 (3.1) | 6 (13) | 5 (42) | 10 (23) | 7 (9.1) | 16 (18) | 23 (14) |
| Left against medical advice | 6 (9.2) | 3 (6.7) | 3 (25) | 11 (26) | 9 (12) | 14 (16) | 23 (14) |
| Died | 2 (3.1) | 1 (2.2) | 0 (0.0) | 0 (0.0) | 2 (2.6) | 1 (1.1) | 3 (1.8) |
| Lost to follow up | 0 (0.0) | 0 (0.0) | 0 (0.0) | 8 (18.6) | 0 (0.0) | 8 (9.1) | 8 (4.9) |
| **bCPAP treatment failure, n (%)** |  | 8 (18) |  | 11 (26) |  | 19 (22) |  |

^a^AKU: Aga Khan University Hospital

^b^ASH: Abbasi Shaheed Hospital
