# Supplementary material for: Reach, implementation fidelity, and safety of bubble continuous positive airway pressure (bCPAP) therapy in children with severe pneumonia in Pakistan
Source: PLOS Glob Public Health. 2026 Jun 15;6(6):e0006232. doi: 10.1371/journal.pgph.0006232 (PMC13268127; doi:10.1371/journal.pgph.0006232)
Supplement: Attachment — Submitted filename: PLOS GPH author response letter 05May2026 final.docx [file pgph.0006232.s006.docx]

**PGPH-D-26-00995** Author Response Letter

Dear Dr. Rebeca Sultana,

Thank you for your continued interest in our manuscript. We are pleased to submit another revised version based on your review. Please find enclosed a revised manuscript as well as a document outlining all changes made in response to the comments and suggested edits. We highlight below our responses to specific comments. We appreciate your continued consideration and look forward to hearing from you soon.

**Editor’s Comments:**

**Journal Requirements:**

**1. Please include a complete copy of PLOS’ questionnaire on inclusivity in global research in your revised manuscript. Our policy for research in this area aims to improve transparency in the reporting of research performed outside of researchers’ own country or community. The policy applies to researchers who have travelled to a different country to conduct research, research with Indigenous populations or their lands, and research on cultural artefacts. The questionnaire can also be requested at the journal’s discretion for any other submissions, even if these conditions are not met. Please find more information on the policy and a link to download a blank copy of the questionnaire here:** [**https://journals.plos.org/globalpublichealth/s/best-practices-in-research-reporting**](https://journals.plos.org/globalpublichealth/s/best-practices-in-research-reporting)**. Please upload a completed version of your questionnaire as Supporting Information when you resubmit your manuscript.**

We have added the completed inclusivity in global research checklist as a supporting information file. We have also added a section at the end of our Methods section referencing this file.

**If the reviewer comments include a recommendation to cite specific previously published works, please review and evaluate these publications to determine whether they are relevant and should be cited. There is no requirement to cite these works unless the editor has indicated otherwise.** 

This is not applicable to the reviewer comments for our manuscript.

**Please review your reference list to ensure that it is complete and correct. If you have cited papers that have been retracted, please include the rationale for doing so in the manuscript text, or remove these references and replace them with relevant current references. Any changes to the reference list should be mentioned in the rebuttal letter that accompanies your revised manuscript. If you need to cite a retracted article, indicate the article’s retracted status in the References list and also include a citation and full reference for the retraction notice.**

We have reviewed the reference list to ensure it is complete and correct. We have not made any changes to our reference list.

**Reviewers' Comments:

Reviewer's Responses to Questions**

**Comments to the Author**
 **1. Does this manuscript meet PLOS Global Public Health’s** [**publication criteria**](https://nam12.safelinks.protection.outlook.com/?url=https%3A%2F%2Ftrack.editorialmanager.com%2FCL0%2Fhttps%3A%252F%252Fjournals.plos.org%252Fglobalpublichealth%252Fs%252Fcriteria-for-publication%2F1%2F010f019dbd15d5c8-10e6ea73-7c89-4f4f-bdf5-fd649bf500e9-000000%2FmbX4rgZ9VI_Z-NjjeeTDt_dlqbSf6U-a_TE1YQx8kAQ%3D258&data=05%7C02%7Cnadir.ijaz%40yale.edu%7C0cbf3851d6d94fe5dd6f08dea20f75f0%7Cdd8cbebb21394df8b4114e3e87abeb5c%7C0%7C0%7C639126384441374354%7CUnknown%7CTWFpbGZsb3d8eyJFbXB0eU1hcGkiOnRydWUsIlYiOiIwLjAuMDAwMCIsIlAiOiJXaW4zMiIsIkFOIjoiTWFpbCIsIldUIjoyfQ%3D%3D%7C0%7C%7C%7C&sdata=eHT7YRmVBb1Eoc3OKJG0siZFG2GUCwNRmylkvuoF90Y%3D&reserved=0)**? Is the manuscript technically sound, and do the data support the conclusions? The manuscript must describe methodologically and ethically rigorous research with conclusions that are appropriately drawn based on the data presented.**

**Reviewer #1: Yes**

**Reviewer #2: Partly**

**2. Has the statistical analysis been performed appropriately and rigorously?**

**Reviewer #1: Yes**

**Reviewer #2: I don't know**

**3. Have the authors made all data underlying the findings in their manuscript fully available (please refer to the Data Availability Statement at the start of the manuscript PDF file)?

The** [**PLOS Data policy**](https://nam12.safelinks.protection.outlook.com/?url=https%3A%2F%2Ftrack.editorialmanager.com%2FCL0%2Fhttps%3A%252F%252Fjournals.plos.org%252Fglobalpublichealth%252Fs%252Fdata-availability%2F1%2F010f019dbd15d5c8-10e6ea73-7c89-4f4f-bdf5-fd649bf500e9-000000%2FdDSCGd-s4cF7GWUNAN_aQyfhgtBIrZSndTPJvjXKhcg%3D258&data=05%7C02%7Cnadir.ijaz%40yale.edu%7C0cbf3851d6d94fe5dd6f08dea20f75f0%7Cdd8cbebb21394df8b4114e3e87abeb5c%7C0%7C0%7C639126384441396536%7CUnknown%7CTWFpbGZsb3d8eyJFbXB0eU1hcGkiOnRydWUsIlYiOiIwLjAuMDAwMCIsIlAiOiJXaW4zMiIsIkFOIjoiTWFpbCIsIldUIjoyfQ%3D%3D%7C0%7C%7C%7C&sdata=aqAwjg0VNDYRFzkurNLsLdoVc%2FudOlk%2BE%2FgEC2tE25Q%3D&reserved=0) **requires authors to make all data underlying the findings described in their manuscript fully available without restriction, with rare exception. The data should be provided as part of the manuscript or its supporting information, or deposited to a public repository. For example, in addition to summary statistics, the data points behind means, medians and variance measures should be available. If there are restrictions on publicly sharing data—e.g. participant privacy or use of data from a third party—those must be specified.**

**Reviewer #1: No**

**Reviewer #2: No**

**4. Is the manuscript presented in an intelligible fashion and written in standard English?

PLOS Global Public Health does not copyedit accepted manuscripts, so the language in submitted articles must be clear, correct, and unambiguous. Any typographical or grammatical errors should be corrected at revision, so please note any specific errors here.**

**Reviewer #1: Yes**

**Reviewer #2: Yes**

**5. Review Comments to the Author

Please use the space provided to explain your answers to the questions above. You may also include additional comments for the author, including concerns about dual publication, research ethics, or publication ethics. (Please upload your review as an attachment if it exceeds 20,000 characters)**

**Reviewer #1:**

**1. The study addresses an important research question to look into potential reasons contributing directly to the outcome of patients on bubble CPAP.**

Thank you for this comment.

**2. You mentioned that bCPAP is the first line of care for severe pneumonia in ASH as per the hospital protocol. How about AKUH? This directly affects the reach.**

Thank you for your comment. AKUH does not have a hospital protocol related to bCPAP. We have now clarified this in the Methods section:

*Lines 105-108: Locally constructed bCPAP (from a sterile saline bottle and nasal cannula interface) has been used at AKUH for over a decade, although AKUH does not have a bCPAP hospital or unit-level protocol or standardized bCPAP staff training. bCPAP-related education is primarily provided informally by experienced staff members.*

We agree that this may directly affect the reach and have added a statement about this to the Discussion:

*Lines 388-391: bCPAP reach was much higher at ASH compared to AKUH. This is likely at least partially due to the absence of a bCPAP protocol at AKUH and its existence at ASH, as well as the presence of high-flow nasal cannula (HFNC) oxygen as an alternative to bCPAP at AKUH, with limited alternative modalities available at ASH.*

**3. Are the clinicians who make the decision to start bCPAP trained on bubble CPAP? This would affect both the reach/adoption and fidelity as well.**

At ASH, clinicians in the emergency department undergo standardized, hands-on bCPAP training, but nurses in the wards do not have standardized training. At AKUH, there is no standardized bCPAP training. We have added this to our description of the two sites in our Methods section:

*Lines 105-107: Locally constructed bCPAP (from a sterile saline bottle and nasal cannula interface) has been used at AKUH for over a decade, although AKUH does not have a bCPAP hospital or unit-level protocol or standardized bCPAP staff training.*

*Lines 118-125: Locally constructed bCPAP (from a sterile saline bottle and a nasal cannula interface) has been used at ASH since the inception of its partnership with the ChildLife Foundation in 2018. ED nurses and doctors (medical officers and resident doctors) receive standardized bCPAP training in accordance with a bCPAP ED protocol. This protocol emphasizes starting bCPAP early for children with tachypnea, respiratory effort, hypoxemia requiring more than 3-4 liters per minute of oxygen, prioritizing infants weighing less than 10kg. There is no such standardized protocol used in the wards, and no formal training provided to ward nurses.*

**4. You mentioned that those who are not physically located were labelled as lost to follow up. You mentioned that the data collection was done on a daily basis mainly from nurse’s report and caregiver’s report which is an important limitation to the study. How was the hospital staff involved in acquiring the required data especially to check the fidelity and minimizing loss to follow up?**

All the children who were lost to follow up were at ASH. Emergency department leadership was involved in data collection to ensure accuracy and to minimize loss to follow up. However, given the clinical load of staff in the wards, we were unable to have a hospital staff member from the wards assist with data collection. This is where children were lost to follow up. We clarify this here:

*Lines 218-221: Throughout the follow-up period, AKUH ED and ward clinical staff and ASH ED leadership assisted RAs with data collection to ensure accuracy and minimize loss to follow up. Due to the census in the ASH wards, clinical staff were unable to assist RAs with data collection.*

As the reviewer mentions, we already acknowledge some of our outcomes being collected by caregiver and nursing report as a limitation in the Discussion section:

*Lines 460-462: Third, we collected data daily, and some of our outcomes, including disruptions in bCPAP therapy and feeding practices, depended on caregiver and nursing report.*

**5. Did you look into the level of monitoring or oversight provided by physicians in both hospitals? The Malawi trial indicated clearly that the monitoring of patients was mainly nurse led with little physician oversight/monitoring which**

Thank you for this point. We did not directly measure the level of oversight provided by physicians in both hospitals. However, based on our observations and knowledge of clinical care at each site (and the bCPAP protocol at ASH), physicians would order and assist with initial bCPAP setup, but nurses would be primarily responsible for bCPAP setup, monitoring, and maintenance. Unlike the Malawi trial, physicians were always available on-site to escalate concerns.

We now clarify this in our descriptions of AKUH and ASH, respectively:

*Lines 108-110: Resident doctors are generally responsible for ordering and setting up bCPAP and are always present on-site, while nurses are responsible for bedside care and bCPAP monitoring and maintenance.*

*Lines 126-128: In both the ED and wards, doctors are responsible for ordering bCPAP and assisting with setup as necessary, and nurses are responsible for bCPAP monitoring and maintenance.*

**6. In your study, caregivers identified majority of bubble CPAP disruption and were important part of resolving the disruption. It would be worth mentioning the importance of caregiver’s engagement in the care of patients on bubble CPAP in your conclusion. This has also been noted in other studies.**

Thank you for this comment. We agree this is an important point and describe this in our Discussion:

*Lines 427-433: Although continuous pulse oximetry is ideal, in settings where this is not feasible, it may be possible to train those who are continuously at the bedside—often caregivers—to recognize bCPAP-related events needing urgent intervention. Indeed, even though they received no formal training, caregivers in our study identified most unplanned disruptions and often worked independently or with nursing staff to resolve the disruption. Future research should explore strategies to educate and involve caregivers in bCPAP-related care to improve safety.*

**Reviewer #2:**

**These are answers to the questions with no as the response,**

**1. I am unable to provide an assessment of the statistical analysis presented by the authors**

**2. The authors do not wish to share their data openly citing that this contains sensitive and potentially identifying information of participants including not having permission from the ethics committees**

Thank you for these comments. As the reviewer stated, because of sensitive and potentially identifying information about the sites and participants, we do not currently have permission to publicly share the dataset.

**Comments to the authors of the manuscript.

The authors have addressed a very pertinent topic and attempted to answer a very relevant question regarding bCPAP for treatment of severe pneumonia in LMICs such as Pakistan.**

Thank you for this comment.

**However a few queries remain regarding the reporting of findings from what was done.**

**1. it is unclear regarding how children from the eligible cohort were selected for bCPAP as it appears that different set of guidelines were used in the two study settings. Specifying how this was done would help clarify the difference in the proportion. Could there have been children who required CPAP where this was withheld due to limitation of resources?**

Yes, as the reviewer stated, each site had a different method of selecting children for bCPAP therapy. We have now clarified this in our description of the sites in the Methods section:

*Lines 105-108: Locally constructed bCPAP (from a sterile saline bottle and nasal cannula interface) has been used at AKUH for over a decade, although AKUH does not have a bCPAP hospital or unit-level protocol or standardized bCPAP staff training. bCPAP-related education is primarily provided informally by experienced staff members.*

*Lines 118-125: Locally constructed bCPAP (from a sterile saline bottle and a nasal cannula interface) has been used at ASH since the inception of its partnership with the ChildLife Foundation in 2018. ED nurses and doctors (medical officers and resident doctors) receive standardized bCPAP training in accordance with a bCPAP ED protocol. This protocol emphasizes starting bCPAP early for children with tachypnea, respiratory effort, hypoxemia requiring more than 3-4 liters per minute of oxygen, prioritizing infants weighing less than 10kg. There is no such standardized protocol used in the wards, and no formal training provided to ward nurses.*

We do not believe bCPAP was withheld at either site due to a limitation of resources, since locally constructed bCPAP was used at both sites, and these materials were not limited at either site.

**2. Was the locally arranged CPAP used in both settings OR was this different? Using different modalities to deliver CPAP would have implications for the 'fidelity', especially regarding the disruptions. This issue needs to be highlighted and also discussed as a limitation if different modes of delivering CPAP was used.**

The same, locally constructed bCPAP setup used at both sites. We have clarified this further in the manuscript text:

*Lines 105-106: Locally constructed bCPAP (from a sterile saline bottle and nasal cannula interface) has been used at AKUH for over a decade*

*Lines 118-120:* *Locally constructed bCPAP (from a sterile saline bottle and a nasal cannula interface) has been used at ASH since the inception of its partnership with the ChildLife Foundation in 2018.*

**3. A significant number of children (16) on bCPAP were transferred to another facility. This has been highlighted only in the supplementary table**

Thank you for this comment. We have now highlighted this in our Table 3 in the main Results section.

**4. What was the reason for the transfer and was this done while children were still on CPAP has not been mentioned anywhere? While authors do explain that these participants were 'followed up till outcome', it is unclear what the outcomes of these children were.**

Thank you for this question. We have added a description of reasons children were transferred to our Methods section:

*Lines 365-369: Of six children transferred to another hospital from AKUH, four were transferred due to bed unavailability, one due to care unaffordability, and one for undocumented reasons. Of ten children transferred to another hospital from ASH, six were transferred due to a need for intensive care, two due to bed unavailability, and two per parental request.*

We specify in our Methods that “Participants were followed up via daily in-person visits until discharge, transfer to another hospital, leaving against medical advice, death, greater than 30 days of admission, or lost to follow-up” (Lines 210-212). These points defined the end of our follow-up period.

**5. it is interesting to note that 'left against medical advice' contributed to the composite outcome of 'treatment failure'. Considering the fact that information for this group of participants was not available following this event, what is the difference between this category and loss to follow up while on bCPAP?**

As the reviewer noted, “left against medical advice” and “loss to follow up” were defined differently. Leaving against medical advice is recorded in and was ascertained from the participants’ medical charts. Loss to follow up was defined by our team’s inability to physically locate or contact a patient, as detailed in our Methods. We have now added a sentence to our Methods to clarify this difference:

*Lines 212-218: At AKUH, participants who could not be physically located in the hospital were deemed lost to follow-up. At ASH, due to high patient volume, RAs requested and used the parent’s or guardian’s contact phone number to follow up participants who could not be physically located in the hospital. For these participants, those without a valid phone number and those who were contacted twice without response were deemed lost to follow up. Participants were deemed to have left against medical advice only if this was recorded in their medical chart.*

Our decision to include “leaving against medical advice” in our composite treatment failure outcome is based on multiple prior trials. We have now expanded on this in our Methods:

*Lines 263-267: Our definition of bCPAP treatment failure was based on similar definitions from prior trials (8,10). As in these prior trials, we included leaving against medical advice in our composite treatment failure outcome so as not to bias the outcome to success in cases where children received bCPAP therapy but left against medical advice before medically ready for discharge.*
